# Evolution by selection, recombination, and gene duplication in MHC class I genes of two Rhacophoridae species

**DOI:** 10.1186/1471-2148-13-113

**Published:** 2013-06-05

**Authors:** Mian Zhao, Yongzhen Wang, Hang Shen, Chenliang Li, Cheng Chen, Zhenhua Luo, Hua Wu

**Affiliations:** 1 Molecular and Behavioural Ecology Research Group, College of Life Sciences, Central China Normal University, 152 Luoyulu, Hongshan District, Wuhan 430079, China

**Keywords:** Major histocompatibility complex, Evolution, *Rhacophorus omeimontis*, *Polypedates megacephalus*

## Abstract

**Background:**

Comparison of major histocompatibility complex (MHC) genes across vertebrate species can reveal molecular mechanisms underlying the evolution of adaptive immunity-related proteins. As the first terrestrial tetrapods, amphibians deserve special attention because of their exposure to probably increased spectrum of microorganisms compared with ancestral aquatic fishes. Knowledge regarding the evolutionary patterns and mechanisms associated with amphibian MHC genes remains limited. The goal of the present study was to isolate MHC class I genes from two Rhacophoridae species (*Rhacophorus omeimontis* and *Polypedates megacephalus*) and examine their evolution.

**Results:**

We identified 27 MHC class I alleles spanning the region from exon 2 to 4 in 38 tree frogs. The available evidence suggests that these 27 sequences all belong to classical MHC class I (MHC Ia) genes. Although several anuran species only display one MHC class Ia locus, at least two or three loci were observed in *P. megacephalus* and *R. omeimontis,* indicating that the number of MHC class Ia loci varies among anuran species. Recombination events, which mainly involve the entire exons, played an important role in shaping the genetic diversity of the 27 MHC class Ia alleles. In addition, signals of positive selection were found in Rhacophoridae MHC class Ia genes. Amino acid sites strongly suggested by program to be under positive selection basically accorded with the putative antigen binding sites deduced from crystal structure of human HLA. Phylogenetic relationships among MHC class I alleles revealed the presence of trans-species polymorphisms.

**Conclusions:**

In the two Rhacophoridae species (1) there are two or three MHC class Ia loci; (2) recombination mainly occurs between the entire exons of MHC class Ia genes; (3) balancing selection, gene duplication and recombination all contribute to the diversity of MHC class Ia genes. These findings broaden our knowledge on the evolution of amphibian MHC systems.

## Background

Genes of the major histocompatibility complex (MHC) encode cell surface glycoproteins that present self and foreign (e.g., pathogens) peptides to T lymphocytes to trigger the appropriate adaptive immune response. These glycoproteins can be divided into two major subgroups: class I and class II MHC molecules, which recognize intracellular and extracellular antigens, respectively. Class I molecules consist of a heavy chain (α chain) and a microglobulin chain (β_2_m chain). The heavy chain has a cytoplasmic region, a trans-membrane region, and three extracellular domains designated α1, α2, and α3 that are encoded by exons 2–4 of the MHC class I genes. The amino acid sites that recognize and bind antigen (antigen binding sites, ABS) are located in domains α1 and α2 [1,2]. Certain MHC class I genes that display a very high level of genetic diversity [3,4] are called classical MHC class I genes or MHC class Ia genes [2]. In contrast, non-classical MHC class I (MHC class Ib) genes possess fewer polymorphisms, are expressed at lower levels in fewer tissues, and perform different functions despite having similar sequences [5,6].

The high level of MHC class I gene polymorphisms is not only due to increased nucleotide and gene diversity [7-9] but also to increased variations on locus numbers [10-12]. More than one MHC class I locus has been observed in many vertebrate categories, and the number of loci varies among species [10,11,13,14]. These polymorphisms are thought to correspond to the function of the MHC because a greater number of polymorphic MHC proteins provide organisms protection against a larger spectrum of pathogens and thus better fitness. In fact, this hypothesis is supported by the correlation between MHC diversity and disease susceptibility observed in many vertebrates [15-17]. Thus, the MHC class I gene is an appropriate genetic marker to study adaptive evolution [17-19].

The evolutionary mechanisms contributing to the diversity of MHC class I genes are of significant interest in the study of MHC evolution. Three main mechanisms have been proposed: (1) balancing selection [19-22], (2) recombination [23-26] and (3) gene duplication [12,13]. (1) An excess of non-synonymous over synonymous substitutions, especially on ABS, is typically detected in MHC class I genes, supporting the occurrence of positive selection [4,27,28]. Due to the coexistence of many relatively frequent alleles at an individual locus, the type of selection that acts on MHC genes is designated balancing selection. Under balancing selection, MHC alleles can be maintained for millions of years; they are passed from ancestral to descendant species such that closely related species with a relatively recent common ancestor present identical or similar alleles. The phylogenetic tree constructed using these alleles revealed that sequences from the same species could have more distant relationships compared with those from different species. This phenomenon is called trans-species polymorphism and is commonly observed in MHC genes [2,29,30]. (2) In many studies, recombination has been shown to be an important evolutionary mechanism responsible for the extremely high level of genetic diversity of MHC class I genes [23-26]. Two types of recombination patterns have been found. One is exon shuffling, which is characterized by recombination between entire exon regions with breakpoints in intronic regions [31]. This type of recombination is common in teleosts [25,32,33], likely due to the presence of repetitive elements in the long introns of teleosts’ MHC genes [33]. The other recombination pattern is mainly observed in mammals and involves exchanges of small fragments of MHC class I genes [34]. (3) Variations in the number of alleles or loci appear when some of the duplicated genes are maintained in the genome for long periods while others become dysfunctional due to deleterious mutations. The mechanism underlying this type of MHC evolution is called the birth-and-death model [35]. It plays an important role in driving the evolution of MHC genes in many vertebrates [12,13,26].

Knowledge on the evolution of MHC class I genes was obtained mainly from studies of mammals, birds, and fish [4,10,18]. Amphibians theoretically deserve special attention while studying MHC evolution because they are the first terrestrial vertebrates, probably facing a very different spectrum and diversity of microorganisms. However, relatively few studies have focused on the evolution of amphibian MHC class I genes, and those few studies have involved a limited number of amphibian species, mainly *Ambystoma mexicanum* in the Caudata [36] and two closely related Pipidae species (*Xenopus lavies* and *X. (Silurana) tropicalis*) in the Anura [37-39]. These studies revealed that (1) Urodela (*Ambystoma*) have multiple MHC class I loci [36], whereas the Anura (Pipidae and Ranidae) have only one [37-41]; it is possible that the number of MHC class I loci increased after the Urodela and Anura but before the Pipidae and Ranidae diverged [40]. (2) Both balancing selection and recombination play an important role in the evolution of MHC class I genes [37,38]; recombination occurred among entire exons, at least in *X. lavies*[37]. (3) Trans-species polymorphisms are common in amphibian MHC class I genes [38]. It is unknown whether the rules revealed by the evolution of a limited number of species can apply to all amphibians. The extent to which *Xenopus* species represent the Anura is limited because the two *Xenopus* species in Pipidae group diverged early from the ancestral anurans [42,43] and live a predominantly aquatic life that differs from the lifestyle of most anurans. A recent study examining six non-model anuran species (family Hylidae: *Agalychnis callidryas*, *Smilisca phaeota*; family Centrolenidae: *Espadarana prosoblepon*; family Ranidae: *Lithobates catesbeianus*, *L. clamitans, L. yavapaiensis*) revealed more complex patterns and mechanisms in the evolution of MHC class I genes [44], which suggests that a greater number of species must be assessed to obtain a more systematic understanding of the evolution of amphibian MHC class I genes. Consequently, studies examining MHC evolution of a greater number of anuran species may be pivotal in better understanding the evolutionary process of acquired immunity in vertebrates.

The Rhacophoridae family includes arboreal species likely facing multifarious microorganisms due to the moist habitat. It belongs to the Ranoidae/Natatanura clade; the Ranidae family is its sister taxon [42]. Here, we selected two Rhacophoridae species, *Rhacophorus omeimontis* and *Polypedates megacephalus*, to conduct the following studies: (1) designing primers to isolate MHC class I genes from the two species; (2) assessing phylogenetic status of the newly isolated genes; (3) identifying the number of MHC class I loci; and (4) screening for signals of natural selection and recombination. The results of this study will provide insight into the evolution of anuran MHC class I genes.

## Methods

### Sampling and RNA extraction

We sampled 11 *P. megacephalus* (all from Sangzhi County, Hunan Province) and 27 *R. omeimontis* (12 from Sangzhi County, Hunan Province and 15 from Baoxing County, Sichuan Province). Total RNA was extracted from liver tissues using TRIzol according to the manufacturer’s recommendations (Invitrogen). All experiments involving live tree frogs were approved by the Animal Ethics Committee at Central China Normal University.

### Primer design

To design appropriate primers to amplify MHC class I genes from our samples, we searched NCBI for published amphibian MHC class I sequences. MHC class I alleles from *A. mexicanum* (AMU83137), *X. laevis/gilli* (AF185583), *Rana temporaria* (FJ385652), *R. pipiens* (AF185587), and *X. ruwenzoriensis* (AF497528) were then compiled and aligned. Based on conserved regions of these sequences, we designed the following primers using Primer3 [45]: forward primer 5’- CTGCGSWAYTATKABACWGCAGTCTC -3’; reverse primer 5’- TYCAGRCTGCTGTGSTCCACAT -3’.

### DNA amplification and cloning

First-strand complementary DNA (cDNA) was synthesized from total RNA using reverse transcriptase M-MLV (TaKaRa) with oligo(dT)_18_ primers and then used as a template for the polymerase chain reaction (PCR) to amplify MHC class I exons 2–4 from our samples. The PCR products were purified using the kit (Promega) and then cloned into the pMD19-T vector (TaKaRa). Recombinant DNA was transformed into TOP-10 *Escherichia coli* cells, which were then plated onto Luria-Bertani (LB) agar and grown overnight at 37°C. Clones were randomly selected to screen for the presence of the inserted DNA fragments by PCR and agarose gel electrophoresis. Approximately 20 colonies with positive transformants were selected for each sample, and each transformant was cultivated separately in LB broth at 37°C for 12 h. The positive clones were sequenced.

Noticeably, we assessed our newly designed primers before amplifying MHC class I genes from large amounts of samples. First, only one sample was randomly selected for RNA extraction, cDNA synthesis, and MHC class I gene amplification, as described above. The sequences of 5 positive clones were blasted on NCBI. These sequences were found to have a very high similarity (>80%) with the published anuran MHC class I genes, which suggests that our primer design was successful.

### Sequence alignment and allele identification

ABI trace files were examined and edited using DNASTAR4. The sequences were then aligned using ClustalW equipped in MEGA5 [46]. Only those sequences that were present in more than one individual were considered alleles and were included in the following analyses.

The newly obtained alleles were translated into amino acid sequences and aligned with human HLA chains with a published three-dimensional structure [47,48]. The structures permitted the identification of key amino acid sites that are likely involved in peptide binding and disulfide bond formation.

### Statistical analysis

The average pairwise nucleotide distances (Kimura-2-parameter model, K2P) and the Poisson-corrected amino acid distances were computed using MEGA5 [46]. Standard errors of the estimates were obtained via 1000 bootstrap replicates.

Several methods were used to detect recombination in our datasets. One method was conducted using the online program GARD (genetic algorithm recombination detection; [49]) at the Datamonkey website (http://www.datamonkey.org/). The other methods, including RDP [50], BOOTSCAN [51,52], GENECONV [53], MAXCHI [54,55], CHIMAERA [55], SISCAN [56], and 3SEQ [57], are all implemented in the program suite Recombination Detection Program version 3 (RDP3; [58]). To minimize the false-positive error rate, the highest acceptable *P* value for inferring recombination events was set at 0.000005 with a window size of 20 nucleotides, and only those breakpoints that were identified using at least four methods were considered valid.

Phylogenetic relationships were constructed using the anuran MHC class I alleles determined herein and those available in NCBI. The compiled, published data included MHC class Ia genes from *A. callidryas*, *S. phaeota*, *E. prosoblepon*, *L. catesbeianus*, *L. clamitans*, *L. yavapaiensis*, and *R. pipiens,* as well as MHC class Ia and Ib genes from *X. tropicalis* and *X. laevis*. To determine the general phylogenetic status of the newly isolated MHC class I genes, MEGA5 was used to construct a neighbor-joining (NJ) tree using the exon 2–4 region of the MHC class I genes [46]. Considering that recombination events can hinder the identification of true phylogenetic relationships, the sequences were then partitioned as individual exons to infer more accurate phylogenetic trees of anuran species MHC class I genes based on the recombination test results. For this procedure, approximately three alleles from each species with available MHC class I gene sequences in NCBI were randomly selected as representatives for inclusion in the tree construction. Selection of the best-fitting models of nucleotide substitution was conducted based on Akaike Information Criterion (AIC) in jModelTest 0.1 [59,60]. Analyses revealed that TPM2uf + G, TrN + G, and TPM3 + I + G were the most appropriate models for exon 2, exon 3, and exon 4, respectively. Bayesian inference trees were then constructed using MrBayes 3.2 [61]. Two independent runs of four Metropolis coupled Markov chain Monte Carlo (MCMC) simulations (three of them ‘heated’, temperature = 0.20) were performed for millions of generations until the standard deviation of the split frequencies was <0.01. The first 25% of the sampled trees from the cold chain were discarded by default. The remaining trees were then used to compute the majority-rule consensus tree and to calculate the posterior probability of each bipartition. In addition, neighbor-joining trees of individual exons were constructed using the matrix of nucleotide distances through MEGA5 [46]. Support for the nodes in the obtained tree was estimated via 1000 bootstrap replicates.

Recombination leads to false inferences of positive selection. Fortunately, partitioning strategy considering separate unrecombinant segments based on deduced recombination breakpoints can largely reduce false positive to acceptable level [62]. We used three approaches all with partitioning pre-analyses to test whether positive selection shaped the evolution pattern of the newly isolated MHC class I sequences. First, the presence of positive selection signals was tested using the Codeml subroutine included in PAML version 4 [63,64]. Maximum likelihood estimations of ω (ω = dN/dS, that is, the ratio of non-synonymous/synonymous substitution rates) among codons were generated using this procedure [65,66]. Six different models corresponding to different distribution patterns of ω were tested in our data: M0 (one ω), M1a (nearly neutral), M2a (positive selection), M3 (discrete); M7 (nearly neutral with the beta distribution approximating ω variation; M8 (positive selection with the beta distribution approximating ω variation) [65-67]. Three likelihood ratio tests (LRTs) were then performed to compare the nested models (M0 vs. M3, M1a vs. M2a, and M7 vs. M8) to test the presence of positive selection in models M2a, M3, and M8. Positively selected sites were identified by Bayes empirical Bayes (BEB) method in models M2a and M8 [67]. However, another two methods for detecting selection signals were also applied considering that the power of Codeml would be affected by the accuracy of the inferred phylogenetic trees [68]. Both methods (FEL and MEME) [69-71] were implemented at the Datamonkey website (http://www.datamonkey.org). A more accurate understanding of the sites affected by positive selection can be obtained by combining the results from all of the present analyses.

## Results

### Allele characterization

**Figure 1:**
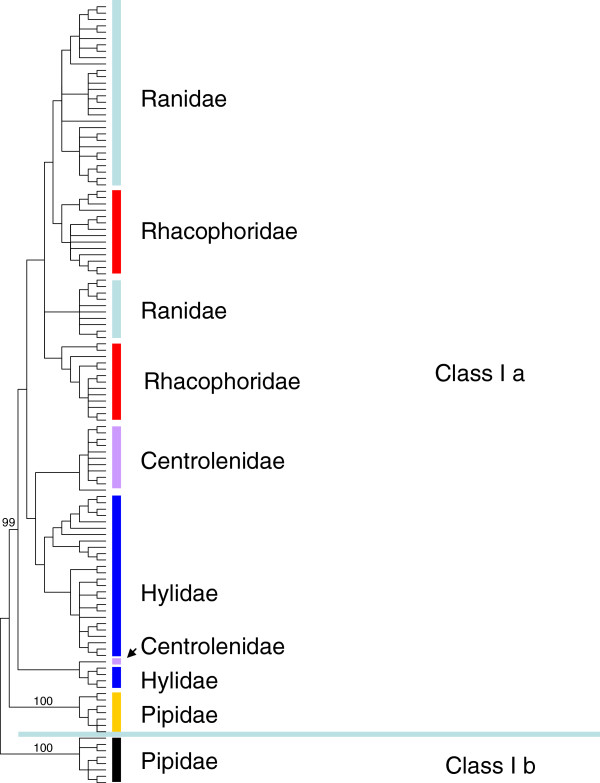
**Phylogenetic relationships of anuran MHC class Ia and Ib genes.** The neighbor-joining tree was constructed with MEGA5 using all available anuran MHC class I genes with a sufficient length. Bootstrap values are indicated above the branches. Sample names are not shown due to the large size of the tree. Rather, the families to which these samples belong are denoted with different colors. The blue horizontal line separates MHC class Ia genes (above the line) and MHC class Ib genes (below the line). Alleles in this tree include the following: the 27 alleles isolated in the present study (family Rhacophoridae); MHC class Ia alleles from the family Hylidae (19 alleles from *A. callidryas*: JQ679312-JQ679330; 11 alleles from *S. phaeota*: JQ679380-JQ679390, [44]); from family Centrolenidae (12 alleles from *E. prosoblepon*: JQ679331-JQ679342, [44]); from family Ranidae (12 alleles from *R. catesbeiana*: JQ679343-JQ679354; 16 alleles from *R. clamitans*: JQ679355-JQ679370; 9 alleles from *R. yavapaiensis*: JQ679371-JQ679379, [44]; 2 alleles from *R. pipiens*: AF185587-AF185588, [72]); from family Pipidae ( 5 alleles from *X. tropicalis*: BC167634, BC161748, BC154904, [73]; NM_001112910, NM_001113065, [74]); 2 alleles from *X. laevis*: (NM_001085732, [72]; NM_001086402, [73]); and MHC class Ib alleles from family Pipidae (6 alleles from *X. laevis*: L20726, L20730, L20732, [6]; FJ589642- FJ589643, [75]; NM_001135072, [72]; 2 MHC Ib alleles from *X. tropicalis*: NM_001037273, NM_001247995, [72]).

Using the techniques described in the Methods, we obtained 757 sequences and finally identified 27 putative MHC class I alleles. These alleles span a portion of exon 2 and of exon 4 and the entire region of exon 3, with a length of 732 nucleotides or 735 nucleotides. Length variation resulting in an indel of one amino acid codon is very common in amphibian species [40,72,73]. Additionally, none of these sequences displayed stop codons. As a result, all of the 27 alleles could be successfully translated into peptides. Comparisons of these peptides with human HLA amino acid sequences revealed that the four pivotal amino acid sites that form disulfide bridges (Cys101-Cys164 and Cys203-Cys259 in human HLA) were conserved in our focal sequences [47,48]. This finding indicated that all of the identified alleles were functional. Next, we constructed a phylogenetic tree using the 27 alleles and all of the published MHC class I genes from anurans with comparable coverage (see Methods) to resolve their phylogenetic status. We found that MHC class Ia genes and MHC class Ib genes clustered separately, and all of the 27 Rhacophoridae alleles belonged to the MHC class Ia gene cluster (Figure 1). This result further suggested that all of the 27 alleles were located at the MHC class Ia locus. We deposited these alleles in GenBank under accession numbers KC261637-KC261663.

### Number of loci

We identified 7 alleles in 11 *P. megacephalus*, 6 alleles in 12 *R. omeimontis* from Hunan, and 14 alleles in 15 *R. omeimontis* from Sichuan. None of these alleles was shared among the three populations. At most three, three, and six alleles were observed respectively in each *P. megacephalus*, *R. omeimontis* from Hunan, and *R. omeimontis* from Sichuan. As *P. megacephalus* and *R. omeimontis* are both diploid [76,77], we concluded that they possess 2 to 3 MHC class Ia loci (Table 1). This result is inconsistent with the findings of previous studies examining two *Xenopus* species and two *Rana* species, from which only one MHC class Ia locus was observed [5,39,40,72,78]. However, it is in accordance with the results of a recent study of 6 non-model anuran species with 2 or 3 MHC class Ia loci [44]. Noticeably, the actual number of loci was probably larger than our prediction because we identified the alleles using a very conservative strategy, and the locus number variation in the two populations of *R. omeimontis* may be a consequence of this underestimation.

**Table 1:** Summary of population allele distributions

	***P. megacephalus *(HN**^**a**^**)**	***R. omeimontis *(HN**^**a**^**)**	***R. omeimontis *(SC**^**b**^**)**
**Sample size**	11	12	15
**No. of alleles**	7	6	14
**Maximal No. of alleles observed in one sample**	3	3	6
**Minimal No. of loci**	2	2	3

Notes: ^a^*HN* Hunan, ^b^*SC* Sichuan.

### Genetic diversity

The MHC class I sequences determined in both species displayed a comparatively level of high genetic diversity similar to other anuran species (Table 2). Exon 2 and the corresponding domain α1 had the highest diversity in both species, while exon 4 and domain α3 had the lowest. This mutation distribution pattern has been observed in other amphibians [44,72] and can be explained by the distinct functions of the different domains. We also noted that there was a lower level of divergence among nucleotide sequences compared with that among amino acid chains. This observation suggests that there were more non-synonymous compared with synonymous mutations, which is consistent with the observations for the sequences under positive selection.

**Table 2:** Average nucleotide and amino acid distances among the 27 newly isolated alleles

	***P. megacephalus***	***R. omeimontis***
**K2P nucleotide distance**	0.156 (0.010)	0.143 (0.009)
Exon 2	0.341 (0.034)	0.319 (0.031)
Exon 3	0.115 (0.014)	0.132 (0.014)
Exon 4	0.069 (0.011)	0.029 (0.006)
**Poisson-corrected amino acid distance**	0.243 (0.023)	0.229 (0.022)
α1	0.486 (0.062)	0.452 (0.056)
α2	0.213 (0.037)	0.240 (0.036)
α3	0.099 (0.026)	0.053 (0.015)

Notes: Standard errors are shown in parentheses.

### Detection of recombination

Using the RDP program, we identified five alleles as recombination products (Table 3). Because we established very conservative parameters to avoid miscalculations, the actual number of recombinants was probably greater than five. However, the five alleles still accounted for a relatively large proportion (18.5%) of these alleles. Using the RDP and GARD programs, four (239, 141, 334, and 229) and three (223, 425, and 531) breakpoints were detected, respectively. Breakpoints located near the boundary of exons 2 and 3 (239, 229, and 223) were found out several times, lending strong support to the presence of recombination via exon shuffling with breakpoints in intron 2. The same type of recombination has been observed in other frogs and bony fishes [25,37,44].

**Table 3:** Recombination test using the RDP program

	**Recombinant sequence**	**Nucleotide breakpoint**	**Potential parental sequences**	**Methods**
1	Rhom16	239	Rhom13/Unknown(Rhom17)	GENECONV, BootScan, MaxChi, Chimaera, Siscan, 3Seq
2	Pome06	239	Rhom15/Rhom18	GENECONV, BootScan, MaxChi, Chimaera, Siscan, 3Seq
3	Rhom17	141	Rhom12/Unknown(Rhom05)	GENECONV, MaxChi, Chimaera, Siscan, 3Seq
4	Pome04	334	Rhom10/Unknown(Rhom12)	MaxChi, Chimaera, Siscan, 3Seq
5	Rhom08	229	Rhom15/Rhom18	RDP, GENECONV, BootScan, MaxChi, Chimaera, Siscan, 3Seq

### Detection of selection

**Figure 2:**
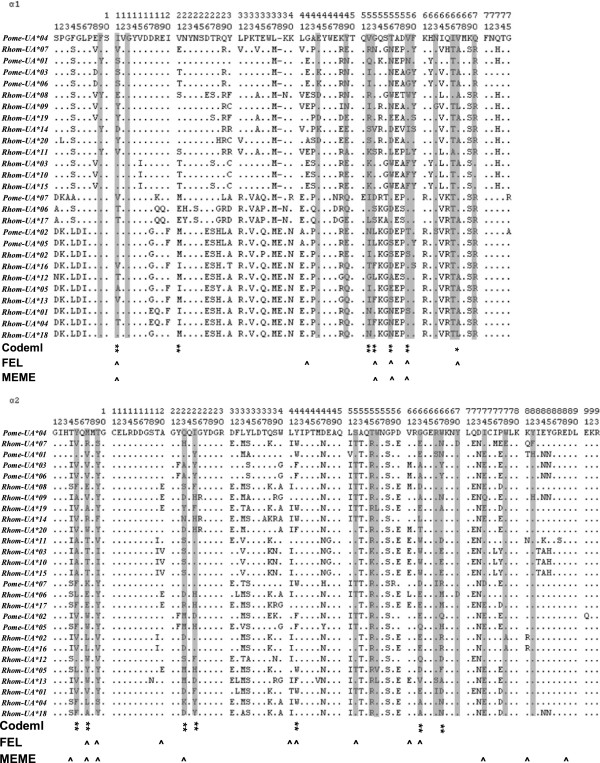
**Alignments of partial amino acid sequences translated from the 27 Rhacophoridae MHC class I alleles.** Dots indicate identity to Pome-UA*04 and signs “-” denote deletions. Sites in shaded columns represent the putative ABS deduced from structural information for human HLA molecules [47,48]. Signs “*”, “**” refer to amino acids predicted to be under positive selection in the M8 model of PAML, with a posterior probability >95% and >99%, respectively. Signs “^” refer to positively selected sites identified by FEL and MEME.

We used three different codon-based maximum likelihood methods all with partitioning pre-analyses to screen for selection signals. Analyses using PAML (Additional files 1 and 2), FEL (Additional file 3), and MEME (Additional file 4) all suggested that Rhacophoridae MHC class Ia alleles underwent natural selection, although the positive selected sites identified by different methods varied (Figure 2 and Table 4). However, the majority of these sites were located on exons 2 and 3 regardless of their differences. In total, 12 out of the 28 sites were identified by more than one program to have undergone positive selection. Excluding two sites on exon 4 and one site on exon 3, the other 9 sites all belonged to the presumed ABS [47,48] (Figure 2). This finding indicated that the Rhacophoridae MHC class I genes underwent pathogen-mediated balancing selection [19].

**Table 4:** Summary statistics for codon sites undergoing positive selection identified by different methods

**Method**	**Sites predicted to be under positive selection**															
α **1**																
	11	21	42	52	53	56	59	67								
Codeml^a^	**	**		**	**	**	**	*								
FEL^b^	^		^		^	^	^	^								
MEME^c^	^				^	^	^									
α **2**																
	4	5	7	9	20	23	25	41	42	52	61	63	67	74	77	81
Codeml^a^		**	**			**	**		**			**	**			
FEL^b^			^	^	^			^	^	^	^	^				
MEME^c^	^		^	^		^								^	^	^
α **3**																
	10	34	40	72												
Codeml^a^	*	*	**	**												
FEL^b^			^	^												
MEME^c^																

Notes: Numbers correspond to the alignment shown in Figure 2. ^a^Amino acids identified in model M8 by the Bayes empirical Bayes procedure [67] using Codeml. Only those sites predicted to be undergoing positive selection with a posterior probability >95% are presented in the table. The * indicates that the posterior probability is >95%, and ** shows that the probability is >99%. ^b^Amino acid sites identified by FEL are identified with ^. ^c^Amino acid sites identified by MEME are identified with ^.

### Phylogenetic analyses

**Figure 3:**
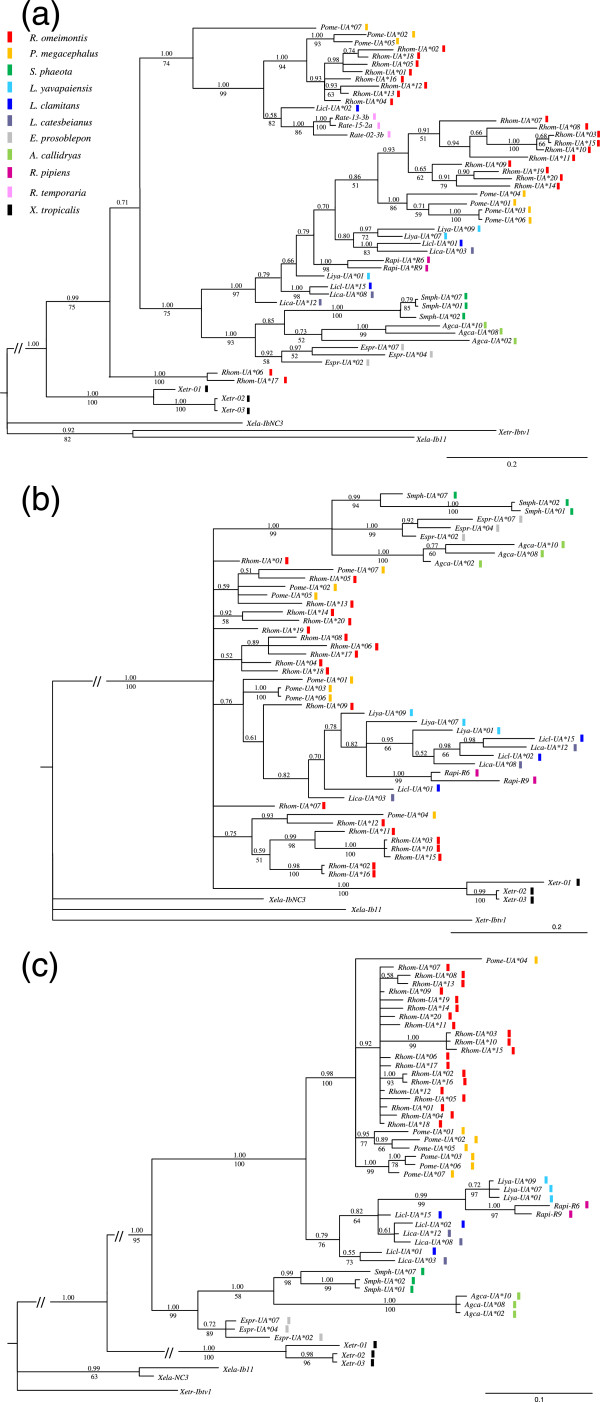
**Phylogenetic relationships of exon 2 (a), exon 3 (b), and exon 4 (c) in anuran MHC class I alleles.** All of the trees were rooted from MHC Ib genes of *Xenopus*. Bootstrap values >50% (from the neighbor-joining analysis) are shown above the respective clades. Bayesian posterior probabilities >0.70 are presented below the clades. All of the branches are proportional to the scale shown at the bottom right of the figure, excluding branches with the sign “//”, which were shortened for graphical clarity of the remaining branches of the tree. Alleles belonging to different species are marked using different colored bars. Involved allelic sequences obtained from NCBI include the following: MHC class Ia alleles from *S. phaeota* (Smph-UA*07: JQ679386; Smph-UA*02: JQ679381; Smph-UA*01: JQ679380); *L. yavapaiensis* (Liya-UA*09: JQ679379; Liya-UA*07: JQ679377; Liya-UA*01: JQ679371); *L. clamitans* (Licl-UA*15: JQ679369; Licl-UA*02: JQ679356; Licl-UA*01: JQ679355); *L. catesbeianus* (Lica-UA*12: JQ679354; Lica-UA*08: JQ679350; Lica-UA*03: JQ679345); *E. prosoblepon* (Espr-UA*07: JQ679337; Espr-UA*04: JQ679334; Espr-UA*02: JQ679332); *A. callidryas* (Agca-UA*10: JQ679321; Agca-UA*08: JQ679319; Agca-UA*02: JQ679313); *R. pipiens* (*Rapi-R6*: AF185587; *Rapi-R9*: AF185588); *R. temporaria* (*Rate-13-3b*: FJ385632; *Rate-02-3b*: FJ385633; *Rate-15-2a*: FJ385641); *X. tropicalis* (*Xetr-01*: BC161748; *Xetr-02*: NM_001113065; *Xetr-03*: BC154904); and MHC Ib alleles from *X. tropicalis* (*Xetr-Ibtv1*: NM_001247995) and from *X.* laevis (*Xela-Ib11*: FJ589643; *Xela-IbNC3*: L20726).

Phylogenetic trees were constructed using available anuran MHC class I genes. Considering that the exon exchanges occurred between exons 2 and 3/4 and that mutation rates were very different between exons 2/3 and 4, we partitioned the aligned sequences into individual exon regions to reconstruct more accurate phylogenetic relationships. In all three trees, the MHC class Ia genes clustered together while remaining distinct from the MHC class Ib genes; the 27 newly obtained alleles clearly belonged to the MHC class Ia gene cluster (Figure 3). The tree constructed for exon 4 was very consistent with species tree [42] because there were five clusters that corresponded well to the anuran taxa: the newly obtained alleles from *P*. *megacephalus* and *R*. *omeimontis* formed the Rhacophoridae cluster with high confidence; alleles from *L. yavapaiensis*, *L. clamitans*, *L. catesbeianus,* and *R. pipiens* formed the Ranidae cluster as the sister clade of Rhacophoridae; alleles from *S. phaeota* and from *A. callidryas* belonged to the Hylidae cluster and then coalesced with alleles from *E. prosoblepon*; alleles from *Xenopus* formed a well-supported clade (Figure 3). In conclusion, exon 4 of the MHC class I genes displayed species-specific clustering. However, in the phylogenetic trees of exons 2 and 3, alleles from the same species did not always display more similarity than those from different taxa. In the tree of exon 2, a portion of the Rhacophoridae alleles branched from common ancestors of Rhacophoridae, Ranidae, Hylidae, and Centrolenidae species; the other portion bifurcated into two clusters. In the tree of exon 3, mixing of alleles from different species could be observed too, although the tree exhibited a star-like topology, likely due to the lack of informative mutations (Figure 3). In conclusion, exons 2 and 3 of Rhacophoridae MHC class I genes possess trans-species polymorphisms [29].

## Discussion

We successfully isolated MHC class I genes from two Rhacophoridae species: *P*. *megacephalus* and *R*. *omeimontis.* Several lines of evidence suggest that all of the newly isolated alleles belong to classical MHC class I loci: (1) the alleles were amplified from total RNA instead of DNA, and therefore, pseudogenes were excluded because they cannot be expressed; (2) neither additional stop codons nor abnormal indels were observed in the alleles; (3) putative amino acids forming disulfide bonds were conserved in all of the alleles; (4) phylogenetic trees of all available anuran MHC class I genes indicated that the alleles isolated in this study clustered together with published MHC class Ia genes while all of the published MHC class Ib genes converged in the other cluster; (5) the alleles showed a high level of genetic diversity and natural selection mainly on the putative ABS.

Based on the observation that as many as three and six alleles were respectively present in individual *P*. *megacephalus* and *R*. *omeimontis* organisms and the knowledge that both of the two Rhacophoridae species are diploid [76,77], we concluded that there were respectively two and three loci in the two Rhacophoridae species. Considering our stringent criterion of allele identification that a sequence was determined as an allele only if it appeared in more than one individual, the actual numbers of MHC class Ia loci in our focal species are probably underestimated due to the removal of alleles with a low frequency. It means that the two Rhacophoridae species express at least two or three MHC class Ia loci. This result was not common for anuran species as previous studies have suggested that the Anura, including two Pipidae species (*X. tropicalis* and *X. laevis*) [5,39] and two Ranidae species (*R. temporaria* and *R. pipiens*) [40,72,78], all expressed one MHC class Ia locus. However, all other vertebrates, including fishes [25], reptiles [79], birds [26] and mammals [12], commonly express more than one locus; a recent study on anuran MHC evolution also revealed that six non-model anuran species all possessed at least two or three MHC class Ia loci [44]. Thus our present study showed two additional anuran species with multiple MHC class Ia loci, supported results of the recent study on anuran MHC class I genes, and presented the ubiquity of multiple MHC class I loci in vertebrates. Our knowledge on amphibian MHC class I genes is updated and broadened by studying more representative species.

**Figure 4:**
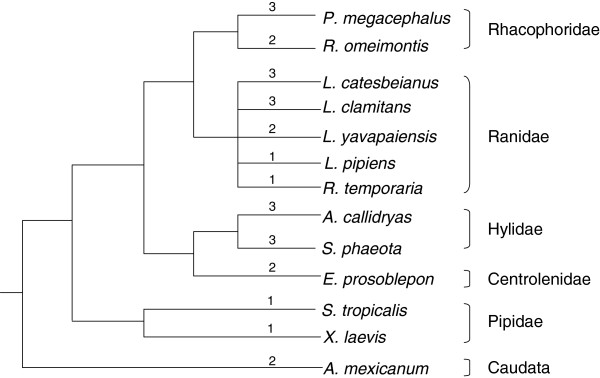
**Minimum numbers of putative MHC class Ia loci in amphibian species.** The simplified phylogenetic tree of amphibian species with a deduced number of MHC class Ia loci is based on the amphibian pedigree shown in [42]. The putative number of loci is shown above the corresponding branches.

High degree of loci number variation has been observed in many vertebrates. For example, Atlantic salmons express three MHC class I loci [80], Chinese sturgeon and paddlefish express two loci [25], while cichlid fishes express 17 loci [81]; chickens have two loci [82] while blue tits express at least 6 loci [26]. For amphibians, previous studies revealed a similar but simple pattern that Anura express one locus [5,39,40,72,78] while Caudata express more than one locus [36]. Based on this limited information, researchers speculated that the diversification of amphibian MHC class I loci numbers occurred sometime after the divergence of Caudata/Anura but before that of Ranidae/Pipidae [40]. However, the present study showed that the numbers of MHC class I loci also varied among the anuran species, which is consistent with one recent study [44]. Number variation of amphibian MHC class I loci is then likely to be more complicated than we thought. After combining all the available information on amphibian MHC class I loci (Figure 4), we found that species belonging to the same family could also have different amounts of loci, suggesting that anuran loci number variation is species specific. This pattern of mixed loci number can be explained by the occurrence of gene duplication/loss during the evolution of amphibian MHC class I genes, that is, the birth-and-death model of evolution [35]. However, this pattern also can be caused by inaccurate estimations of loci number in each species. The two causes are not incompatible. Further analyses to examine accurate number of anuran MHC class Ia loci can help to understand the complete history of anuran gene duplications.

Interestingly, we even observed loci number variation in two geographic populations of the same species (*R*. *omeimontis*) (Table 1)*.* However, the observation either mirrors actual loci number variation or just results from underestimation of loci number. We try to distinguish the two causes based on the experiences that MHC alleles from the same locus tended to cluster together [83,84]. Phylogenetic tree constructed from exon 2 sequences of anuran MHC class I alleles (Figure 3a) was chosen as reference because this tree had a clear topology due to high polymorphic sequences and had high veracity due to the partitioning strategy. We found that 20 alleles from *R. omeimontis* formed three distant clusters. Alleles both from Hunan population (Rhom-UA*01 to Rhom-UA*06) and from Sichuan population (from Rhom-UA*07 to Rhom-UA*20) distributed in the three clusters. This pattern suggests that the two populations of *R*. *omeimontis* both have three MHC class I loci, and the observed loci number variation is caused by underestimation of loci number in Hunan population*.* Previous studies have also revealed locus number variations among individuals or populations of the same species [13,85]. Similarly, it is early to take the variations as truth before further analyses were performed to preclude the possibility of loci number underestimation.

Recombination is one of the most important factors driving evolution of MHC class I genes [23-26]. In the present study, recombination was found to play an important role in the evolution of Rhacophoridae MHC class Ia genes. 18.5% of the alleles were generated by recombination, and the percentage is likely an underestimation of the actual value because the alleles were identified using very conservative criteria. Several breakpoints were identified, and a large portion of them were located near the boundary of exons 2 and 3. This finding indicates that the exchange of DNA occurred mainly between exons 2 and 3/4. The recombination pattern is congruent with that mainly observed in the MHC class I genes of bony fishes and other anurans [24,25,37], but different from observations in mammals which have reticulate recombination pattern [34]. It suggests that the MHC class I gene of anuran and of teleostean might have some similar gene organization contributing to the same exon shuffling pattern. Previous studies have revealed that intron 2 of MHC class I genes in bony fishes exhibits very long coverage and contains numerous repeated elements [33]. The two characters of gene organization were regarded to be pivotal to make intron 2 a recombination hot spot region. However, the intron 2 sequences of Rhacophoridae MHC class I genes were not available for analysis. We deduce that Rhacophoridae species likely also have a large intron 2 region in their MHC class I genes based on the genetic information obtained from the whole genome of *X. tropicalis*[37]. Certainly, additional studies specifically on MHC gene organization are required if we want to confirm this deduction.

Several evidence suggests that Rhacophoridae MHC class I genes have undergone natural selection. First, the nucleotide sequence divergence of these genes was reduced compared with the amino acid sequence divergence, consistent with the positive selection signal showing that non-synonymous were more frequent than synonymous variations. Second, several codon-based methods have detected natural selection signals as well as the sites probably under positive selection in the 27 MHC class I alleles. The sites that were identified by more than one method were considered robust; they belonged to the subset of putative ABS deduced from the crystal structure of human HLA [47,48], with three exceptions. This result indicated that the structures of Rhacophoridae class I MHC molecules were similar to that of human HLA despite some differences. Similar results have been observed in many other species [25,38]. Considering the function of ABS, we conclude that the MHC class I genes identified herein are most likely undergoing pathogen-mediated balancing selection.

Trans-species polymorphisms of anuran MHC class Ia genes were evident considering that MHC class Ia alleles from Ranidae, Racophoridae, Hylidae, and Centrolenidae all shared common ancestry (Figure 1). More specifically, the trans-species polymorphisms were observed in exons 2 and 3 of anuran MHC class I genes (Figure 3). When constructing NJ trees using synonymous mutations, we did not observe species specific topology in the phylogenetic trees of exons 2 and 3 (data not shown). This finding precludes the possibility that concerted evolution caused the trans-species polymorphism. Then balancing selection was most likely to be correlated with the trans-species polymorphism pattern. Specifically speaking, α1 and α2, which contain key regions responsible for antigen recognition and binding, undergo strong balancing selection such that the nucleotide sequences encoding these domains can be maintained for long periods. With sufficient persistence, the origin of the alleles predates the species divergence. In contrast, exon 4 encoding α3, which functions as an anchor and a linker, suffers much fewer selection constraints. Consequently, the pedigree of exon 4 commonly reflects accurate phylogenetic relationships of these species. The difference in evolution patterns for different exons of the same gene has been observed in previous studies [25,44,84].

In trans-species polymorphisms, mixing of alleles from different species results from the long-time persistence of MHC genes which originate before births of the corresponding species [29]. So we can deduce the time when MHC genes come into being according to divergence time of related species. In the present study, two anuran MHC class Ia allelic lineages were likely to originate respectively before divergence of Ranidae/Racophoridae and that of Hylidae/Centrolenidae (Figure 3a). As these two divergence events respectively date back to 69–72 and 68–91 million years ago (MYA) [43], the oldest MHC class Ia allelic lineages for Anura was estimated to originate about 70 MYA. This time to maintain MHC class I alleles for Anura is considerably long compared to that for some other vertebrates [86]. For example, the oldest allelic lineages at human HLA loci A, B and C was deduced to diverge about 18, 22 and 10 MYA, respectively [29].

## Conclusion

In the present study, we designed new primers to successfully isolate MHC class Ia genes from two Rhacophoridae species (*P. megacephalus* and *R. omeimontis*). Both species were presumed to contain two or more MHC class Ia locus. Frequent gene duplications, recombination mainly due to exchanges of whole exons, and balancing selection predominantly acting on the ABS, were all considered to play an important role in the evolution of Rhacophoridae MHC class I genes. The current results broaden our knowledge of the evolution of anuran MHC genes.

## Abbreviations

MHC: Major histocompatibility complex; cDNA: Complementary DNA; PCR: Polymerase chain reaction; ABS: Antigen binding sites; MYA: Million years ago.

## Competing interests

The authors declare no competing interests.

## Authors’ contributions

MZ performed the data analysis and drafted the manuscript; YW, HS and CC carried out the molecular experiments; CL performed collection of samples; ZL participated in the data analysis; HW designed the study, participated in the data analysis and helped to draft the manuscript. All authors read and approved the final manuscript.

## Supplementary Material

Additional file 1 **Goodness of fit for different codon evolution models and estimated parameter values.** Notes: P refers to the number of parameters in the x distribution; x is the selection parameter. pn is the proportion of sites falling within the xn site class. For models M7 and M8, p and q denote the shape parameters of the b function. Positively selected sites were identified in models M2a and M8 using the Bayes empirical Bayes procedure [67]. Sites inferred to be undergoing positive selection at the 95% and 99% confidence interval level are marked with * and **, respectively. Click here for file

Additional file 2 **Summary of the test statistics for the likelihood ratio test of codon evolution.** Notes: df refers to degrees of freedom. Test statistics were computed using formula 2 (Lb - La); La and Lb are log-likelihood values for each of the nested models being compared. Click here for file

Additional file 3 **Sites undergoing positive selection detected using the FEL program.** Note: Numbering of the three domains differs from that shown in Additional file 1. Rather, the sites positions of domains α2 and α3 are counted from the first amino acids of domain α1. Only those sites with a significance level <0.05 are shown. ^a^Normalized dN-dS refers to dN-dS divided by the total length of the appropriate tree. Click here for file

Additional file 4 **Sites undergoing positive selection detected using the MEME program.** Note: The numbering strategy is described in Additional file 3. This summary table reports the distribution of synonymous (α) and non-synonymous (β) substitution rates for sites inferred using the MEME model. Sites with a significance level <0.01 are shown. Click here for file
