# Supplementary material for: Evolution by selection, recombination, and gene duplication in MHC class I genes of two Rhacophoridae species
Source: BMC Evol Biol. 2013 Jun 5;13:113. doi: 10.1186/1471-2148-13-113 (PMC3684511; doi:10.1186/1471-2148-13-113)
Supplement: Additional file 1 — Goodness of fit for different codon evolution models and estimated parameter values. Notes: P refers to the number of parameters in the x distribution; x is the selection parameter. pn is the proportion of sites falling within the xn site class. For models M7 and M8, p and q denote the shape parameters of the b function. Positively selected sites were identified in models M2a and M8 using the Bayes empirical Bayes procedure [67]. Sites inferred to be undergoing positive selection at the 95% and 99% confidence interval level are marked with * and **, respectively. [file 1471-2148-13-113-S1.doc]

Additional file 1. Goodness of fit for different codon evolution models and estimated parameter values.

| **Model code** | **P** | **Log-likelihood** | **Parameter estimates** | **Positively selected sites** |
| --- | --- | --- | --- | --- |
| **α 1** |  |  |  |  |
| M0 | 1 | -2206.747674 | w = 0.744, K =2.066 |  |
| M1a | 1 | -2129.325007 | p0 = 0.601, p1 = 0.399, K = 1.829, w0 = 0.151, w1 = 1.000 |  |
| M2a | 3 | -2092.918983 | p0 = 0.506, p1 = 0.379, p2 = 0.115, K = 2.217, w0 = 0.172, w1 = 1.000, w2 =4.384 | 11I** 21V** 52V** 53G* 56T** 59V** 67V |
| M3 | 5 | -2092.690588 | p0 = 0.551, p1 = 0.357, p2 = 0.092, K = 2.195, w0 = 0.200, w1 = 1.205, w2 = 5.136 |  |
| M7 | 2 | -2134.336033 | p = 0.386, q = 0.433, K = 1.805 |  |
| M8 | 4 | -2093.835677 | p0 = 0.866, p1 = 0.134, p2 =0.561, q =0.642, w = 3.949, K =2.222 | 11I** 21V** 30Y 49Y 52V** 53G** 56T** 58D 59V** 60F 67V* |
| **α 2** |  |  |  |  |
| M0 | 1 | -2328.178394 | w = 1.266, K =0.885 |  |
| M1a | 1 | -2157.444325 | p0 = 0.586, p1 = 0.414, K = 0.611, w0 = 0.031, w1 = 1.000 |  |
| M2a | 3 | -2078.742870 | p0 = 0.544, p1 = 0.360, p2 = 0.095, K = 0.782, w0 = 0.034, w1 = 1.000, w2 =6.141 | 5Y** 7M** 23Q** 25I** 34Y 42Y** 63G** 67W** |
| M3 | 5 | -2064.634514 | p0 = 0.569, p1 = 0.352, p2 = 0.079, K = 0.982, w0 = 0.081, w1 = 2.662, w2 = 13.721 |  |
| M7 | 2 | -2158.848676 | p = 0.120, q = 0.194, K = 0.567 |  |
| M8 | 4 | -2081.971407 | p0 = 0.901, p1 = 0.099, p2 =0.018, q =0.023, w = 6.220, K =0.790 | 5Y** 7M** 23Q** 25I** 34Y 40W 42Y** 63G** 67W** 77P |
| **α 3** |  |  |  |  |
| M0 | 1 | -862.485655 | w = 0.672, K = 4.540 |  |
| M1a | 1 | -843.939513 | p0 = 0.645, p1 = 0.355, K = 4.101, w0 = 0.059, w1 = 1.000 |  |
| M2a | 3 | -835.206032 | p0 = 0.821, p1 = 0, p2 = 0.179, K =4.676, w0 = 0.192, w1 = 1.000, w2 =3.261 | 10R 16A 34K 40V* 42H 61M 71S 72V** 74N |
| M3 | 5 | -835.206032 | p0 = 0.339, p1 = 0.482, p2 = 0.179, K = 4.676, w0 = 0.192, w1 =0.192, w2 = 3.261 |  |
| M7 | 2 | -844.342894 | p = 0.012, q = 0.015, K = 4.103 |  |
| M8 | 4 | -835.222427 | p0 = 0.821, p1 = 0.179, p2 = 23.778, q = 99.000, w = 3.266, K = 4.676 | 10R* 16A 34K* 40V** 42H 44L 61M 71S 72V** 74N |

Notes: P refers to the number of parameters in the x distribution; x is the selection parameter. pn is the proportion of sites falling within the xn site class. For models M7 and M8, p and q denote the shape parameters of the b function. Positively selected sites were identified in models M2a and M8 using the Bayes empirical Bayes procedure [67]. Sites inferred to be undergoing positive selection at the 95% and 99% confidence interval level are marked with * and **, respectively.
