# Supplementary material for: Evolution by selection, recombination, and gene duplication in MHC class I genes of two Rhacophoridae species
Source: BMC Evol Biol. 2013 Jun 5;13:113. doi: 10.1186/1471-2148-13-113 (PMC3684511; doi:10.1186/1471-2148-13-113)
Supplement: Additional file 2 — Summary of the test statistics for the likelihood ratio test of codon evolution. Notes: df refers to degrees of freedom. Test statistics were computed using formula 2 (Lb - La); La and Lb are log-likelihood values for each of the nested models being compared. [file 1471-2148-13-113-S2.doc]

Additional file 2. Summary of the test statistics for the likelihood ratio test of codon evolution.

| **Region** | **Models compared** | **df** | **Test statistic** | **Significance (*P*)** |
| --- | --- | --- | --- | --- |
| **α 1** | M3 vs. M0 | 4 | 228.114 | <0.001 |
|  | M2a vs. M1a | 2 | 72.812 | <0.001 |
|  | M8 vs. M7 | 2 | 81.001 | <0.001 |
| **α 2** | M3 vs. M0 | 4 | 527.088 | <0.001 |
|  | M2a vs. M1a | 2 | 157.403 | <0.001 |
|  | M8 vs. M7 | 2 | 153.755 | <0.001 |
| **α 3** | M3 vs. M0 | 4 | 54.559 | <0.001 |
|  | M2a vs. M1a | 2 | 17.467 | <0.001 |
|  | M8 vs. M7 | 2 | 18.241 | <0.001 |

Notes: df refers to degrees of freedom. Test statistics were computed using formula 2 (Lb - La); La and Lb are log-likelihood values for each of the nested models being compared.
