# Supplementary material for: Evolution by selection, recombination, and gene duplication in MHC class I genes of two Rhacophoridae species
Source: BMC Evol Biol. 2013 Jun 5;13:113. doi: 10.1186/1471-2148-13-113 (PMC3684511; doi:10.1186/1471-2148-13-113)
Supplement: Additional file 3 — Sites undergoing positive selection detected using the FEL program. Note: Numbering of the three domains differs from that shown in Additional file 1. Rather, the sites positions of domains α2 and α3 are counted from the first amino acids of domain α1. Only those sites with a significance level <0.05 are shown. aNormalized dN-dS refers to dN-dS divided by the total length of the appropriate tree. [file 1471-2148-13-113-S3.doc]

Additional file 3. Sites undergoing positive selection detected using the FEL program.

| **Codon** | **dS** | **dN** | **dN/dS** | **Normalized dN-dS ^a^** | **p-value** |
| --- | --- | --- | --- | --- | --- |
| 11 | 0.531 | 8.998 | 16.944 | 6.318 | 0.004 |
| 42 | 5.000E-09 | 2.038 | 407572000.000 | 1.521 | 0.032 |
| 53 | 2.708 | 10.193 | 3.764 | 5.585 | 0.049 |
| 56 | 5.000E-09 | 14.983 | 2996600000.000 | 11.180 | 0.002 |
| 59 | 0.690 | 6.924 | 10.039 | 4.652 | 0.007 |
| 67 | 0.869 | 5.683 | 6.539 | 3.592 | 0.023 |
| 82 | 0.289 | 10.992 | 38.081 | 7.987 | 0.008 |
| 84 | 5.000E-09 | 4.109 | 821760000.000 | 3.066 | 0.021 |
| 95 | 5.000E-09 | 2.246 | 449168000.000 | 1.676 | 0.041 |
| 116 | 5.000E-09 | 2.358 | 471548000.000 | 1.759 | 0.040 |
| 117 | 5.000E-09 | 6.587 | 1317412000.000 | 4.915 | 0.024 |
| 127 | 5.000E-09 | 2.839 | 567748000.000 | 2.118 | 0.019 |
| 136 | 5.000E-09 | 2.714 | 542752000.000 | 2.025 | 0.029 |
| 138 | 5.000E-09 | 7.681 | 1536116000.000 | 5.731 | 0.037 |
| 208 | 5.000E-09 | 1.903 | 380616000.000 | 1.420 | 0.028 |
| 240 | 5.000E-09 | 2.555 | 510932000.000 | 1.906 | 0.040 |

Note: Numbering of the three domains differs from that shown in Additional file 1. Rather, the sites positions of domains α2 and α3 are counted from the first amino acids of domain α1. Only those sites with a significance level <0.05 are shown. ^a^Normalized dN-dS refers to dN-dS divided by the total length of the appropriate tree.
