# Supplementary material for: Evolution by selection, recombination, and gene duplication in MHC class I genes of two Rhacophoridae species
Source: BMC Evol Biol. 2013 Jun 5;13:113. doi: 10.1186/1471-2148-13-113 (PMC3684511; doi:10.1186/1471-2148-13-113)
Supplement: Additional file 4 — Sites undergoing positive selection detected using the MEME program. Note: The numbering strategy is described in Additional file 3. This summary table reports the distribution of synonymous (α) and non-synonymous (β) substitution rates for sites inferred using the MEME model. Sites with a significance level <0.01 are shown. [file 1471-2148-13-113-S4.doc]

**Additional file 4. Sites undergoing positive selection detected using the MEME program.**

| Codon | α | β^-^ | Pr [β=β^-^] | β^+^ | Pr [β=β^+^] | p-value | q-value |
| --- | --- | --- | --- | --- | --- | --- | --- |
| 窗体顶端  11 | 0.432 | 0.432 | 0.232 | 12.110 | 0.768 | 0.006 | 0.150 |
| 53 | 2.142 | 0.446 | 0.562 | 36.597 | 0.438 | 0.001 | 0.062 |
| 56 | 0.000 | 0.000 | 0.541 | 53.015 | 0.459 | 8.561E-05 | 0.007 |
| 59 | 0.622 | 0.000 | 0.688 | 28.646 | 0.312 | 0.000 | 0.006 |
| 79 | 3.242 | 0.191 | 0.854 | 10000.000 | 0.146 | 0.002 | 0.084 |
| 82 | 0.000 | 0.000 | 0.363 | 19.807 | 0.637 | 0.006 | 0.164 |
| 84 | 0.000 | 0.000 | 0.703 | 16.442 | 0.297 | 0.005 | 0.142 |
| 98 | 0.000 | 0.000 | 0.723 | 148.068 | 0.277 | 3.311E-05 | 0.004 |
| 149 | 0.000 | 0.000 | 0.842 | 252.466 | 0.158 | 6.060E-09 | 1.485E-06 |
| 152 | 3.417 | 0.129 | 0.782 | 114.716 | 0.218 | 0.002 | 0.082 |
| 156 | 0.000 | 0.000 | 0.849 | 17.809 | 0.151 | 0.007 | 0.148 |

Note: The numbering strategy is described in Additional file 3. This summary table reports the distribution of synonymous (α) and non-synonymous (β) substitution rates for sites inferred using the MEME model. Sites with a significance level <0.01 are shown.
